# COVID-19, Quarantines, Sheltering-in-Place, and Human Rights: The Developing Crisis

**DOI:** 10.4269/ajtmh.20-0528

**Published:** 2020-06-05

**Authors:** John J. Openshaw, Mark A. Travassos

**Affiliations:** 1 Stanford University, Stanford, California;; 2 University of Maryland School of Medicine, Baltimore, Maryland

## Abstract

As COVID-19 has spread across the globe, quarantines and sheltering-in-place orders have become important public health tools but, as currently implemented, have eroded human rights, particularly for the marginalized, including essential workers, detainees, women, and children. Quarantines and sheltering-in-place orders must include explicit guarantees of human rights protections. We outline protections for the quarantined that communities and governments should strive to guarantee.

Following the detection of COVID-19 infection on the *Diamond Princess* cruise ship in early February, the Japanese government imposed a quarantine: all remaining passengers were to remain in their quarters. Whereas guests sheltered in their rooms, the crew continued their daily routines, preparing and delivering meals to guests. Crew members ate communal meals of up to 12 at a table and shared quarters and toilets—even after some were diagnosed with COVID-19.^1^

The imposition of quarantine solely for passengers, not crew, had disastrous consequences. Over the next 2 weeks, 705 additional cases of COVID-19 were diagnosed among the 3,711 passengers and crew. Eventually, more than 10% of the 1,045 crew were diagnosed with COVID-19. Japanese health officials have now admitted that the quarantine aided the spread of disease.^2^

As COVID-19 has spread, similar quarantines have followed in its wake. Quarantine and isolation represent long-standing public health practices aimed at halting disease spread.^3^ Quarantine aims to limit exposed, potentially infected individuals to movement within a prescribed space. Given the limitations of disease detection and testing, infectious and susceptible individuals might be confined together. By contrast, “isolation” refers to the separation of individuals who are known to be infected. “Quarantine” is what occurred on the *Diamond Princess* and the *Grand Princess* cruise ships. A sheltering-in-place order represents the most basic effort of many governments to restrict movement and contain COVID-19 spread. Globally, at least three billion people currently face such restrictions of their activities.^4^ In the hardest hit COVID-19 regions, including Wuhan, China; Bergamo, Italy; and Igualada, Spain, a more restrictive order has been used, with governments prohibiting movement in and out, reviving the practice of a “cordon sanitaire.” Quarantines and sheltering-in-place orders represent a few of the limited public health tools available to protect the greater population, particularly given the lack of a vaccine, effective treatment, or adequate testing.

But who protects the quarantined? Waxing and waning of restrictions on movement may soon become a reality, especially as testing and non-pharmaceutical interventions are heterogeneously applied over geographical space. Restriction orders have the potential to protect the public against illness, but those that benefit most are often outside the boundaries of restricted areas, and those within can suffer deadly consequences as inequalities persist. How do we ensure that lengthy isolation does not interrupt the care of the sick, the safeguarding of the uninfected, and the protection of the most vulnerable? As presently manifested, COVID-19 quarantines and sheltering-in-place orders have demonstrated threats to several vulnerable populations, as detailed in the following text.

1. Essential workers: Quarantines and sheltering-in-place orders have placed an undue burden on essential workers. An early sign of this was on quarantined cruise ships, where workers put their health at risk, providing essential services. Essential workers in industries such as health care, transportation, and food processing and distribution have similarly suffered. Across the United States, meatpacking facilities–deemed by the federal government to be essential infrastructure–have been tied to more than 16,000 COVID-19 cases and 64 worker deaths.^5^ At least 5,500 U.S. grocery store workers have contracted and at least 100 died from COVID-19.^6^ Strict quarantines increase the burden on healthcare workers, staffing hospitals in challenging conditions with limited resources. In the cordon sanitaire that Spain imposed around Igualada, a regional COVID-19 epicenter, the eventual outbreak included 154 COVID-19–positive healthcare workers, placing a strain on the regional hospital, as a third of hospital employees were sent home.^7^ Compounding such difficult situations, some hospitals and health systems have punished healthcare workers who speak out about working under these conditions.^8^
2. Detainees: Some of the most vulnerable quarantined populations include the defenseless: the homeless, prisoners, and detainees, particularly migrants and asylum seekers held in crowded conditions without provision for social distancing. In recent weeks, COVID-19 has spread through these populations, including detained, unaccompanied migrant children.^9^ There have been more than 1,100 confirmed COVID-19 cases in the U.S. Department of Homeland Security’s Immigration and Customs Enforcement facilities.^10^ Advocacy for these populations has been underwhelming. As COVID-19 spread throughout Ohio, Governor Mike DeWine recommended early release for just 38 of 48,891 prisoners.^11^ The federal government has focused its attention on closing the southern border, ignoring the tinderbox conditions faced by current migrant and asylum detainees.^12^
3. Women and children: Quarantines and sheltering-in-place orders have amplified the difficulties faced by women and children trapped in dangerous living situations with their abusers. At the same time, quarantines have placed a strain on the resources available to them, as courts close and shelters must limit intake to avoid overcrowding.^13^

Quarantines and sheltering-in-place orders will continue to be essential tools for containing COVID-19. To better protect vulnerable populations, these practices must acknowledge the rights of individuals as outlined in the Universal Declaration of Human Rights.^14^ The UN General Assembly adopted this declaration in 1948; it has a striking relevance now as we struggle to balance the risks of social contact versus isolation. In the following text, we describe several protections that should be guaranteed for populations sheltering in place, quarantined, or isolated and indicate their basis in the Universal Declaration’s articles (Table 1).

1. Right to a safe workplace (Article 23): Employers must provide provisions for a safe workplace and housing that minimize the risk of transmission, including separation of infected and exposed staff, personal protective equipment as needed, and access to and financial support for medical treatment when infected or exposed.
2. Protections for the vulnerable (Article 25): Detained individuals have the right to live in conditions where they can observe social distancing. They have the right to conditions that protect against the spread of disease, including basic sanitation such as soap and water, clean bedding, and access to medical care. In the event of infection within a detainment center, detainees with COVID-19 should be isolated to minimize risk to the uninfected. Detainees exposed to COVID-19 deserve the right to prompt medical evaluation and care and should not be cohorted with detainees known to be infected with COVID-19. Every attempt should be made to give homeless individuals a safe place to stay while sick or infectious.
3. Right to safe shelter (Article 25): Governments must protect the safety of the most vulnerable who shelter in place, and resources must be made available to guarantee women and children the right to safe shelter, alternative housing, and legal resources and counsel.
4. Right to medical treatment and testing (Article 25): Within the capacity of the healthcare system, restrictions on movement should not limit testing and healthcare services. Needed medical equipment and supplies should safely and quickly move into quarantined areas, and medical treatment, including mental health services, should be accessible.
5. Right to communication, good information, and good counsel (Article 19): Communication should remain open, epidemiologic data should be shared, and expert advice should be widely available. Governments, traditional media, and social media companies have the responsibility to vet information sources and not spread misinformation.

**Table 1:** Relevant articles from the Universal Declaration of Human Rights^14^

Article 19.
Everyone has the right to freedom of opinion and expression; this right includes freedom to hold opinions without interference and to seek, receive and impart information and ideas through any media and regardless of frontiers.
Article 23.
(1) Everyone has the right to work, to free choice of employment, to just and favourable conditions of work and to protection against unemployment.
(2) Everyone, without any discrimination, has the right to equal pay for equal work.
(3) Everyone who works has the right to just and favourable remuneration ensuring for himself and his family an existence worthy of human dignity, and supplemented, if necessary, by other means of social protection.
(4) Everyone has the right to form and to join trade unions for the protection of his interests.
Article 25.
(1) Everyone has the right to a standard of living adequate for the health and well-being of himself and of his family, including food, clothing, housing and medical care and necessary social services, and the right to security in the event of unemployment, sickness, disability, widowhood, old age or other lack of livelihood in circumstances beyond his control.
(2) Motherhood and childhood are entitled to special care and assistance. All children, whether born in or out of wedlock, shall enjoy the same social protection.

We recognize that, like the Universal Declaration of Human Rights itself, some of these protections are aspirational. Given the rapid spread of COVID-19, even the most developed healthcare systems and governments may not be able to provide all services needed. The barriers to providing these services in low-income countries will be exceedingly difficult to surmount. But, despite these challenges, health, governmental, and human rights professionals should strive to codify these protections. Such measures are essential for a humane response to one of the greatest health threats of the twenty-first century. Now is the time for action to protect the most vulnerable.
